# Magnitude and predictors of common mental disorders among residents in south Gondar Zone, Northwest Ethiopia: a community-based, cross-sectional study

**DOI:** 10.1186/s12888-022-03966-4

**Published:** 2022-05-05

**Authors:** Getasew Legas, Getnet Mihretie Beyene, Sintayehu Asnakew, Amsalu Belete, Shegaye Shumet, Nigusie Selomon Tibebu, Ermias Sisay Chanie, Agimasie Tigabu, Moges Wubneh Abate, Adane Birhanu Nigat, Tigabu Munye

**Affiliations:** 1 Department of Psychiatry, College of Medicine and Health Sciences, Debre Tabor University, Debre Tabor, Ethiopia; 2 Department of Psychiatry, College of Medicine and Health Sciences, University of Gondar, Gondar, Ethiopia; 3 Department of Adult Health Nursing, College of Health Sciences, Debre Tabor University, Debre Tabor, Ethiopia; 4 Department of Pediatrics and Child Health Nursing, College of Health Science, Debre Tabor University, Debre Tabor, Ethiopia

## Abstract

**Background:**

Common mental disorders such as depression, anxiety, and somatic symptoms are a major public health concern because it is prevalent and chronic, and its impact on physical health, psychological and economic consequences is very serious. Evidence on the prevalence and predictors of common mental disorders is very limited in Ethiopia. This study aims to determine the prevalence and associated factors with common mental disorders.

**Methods:**

A community-based cross-sectional study was conducted among 731 south Gondar zone residents recruited with a multistage sampling method. Data were collected by face-to-face interviews on socio-demographic, clinical, and psychosocial factors. Common mental disorders (CMD) were assessed using a self-reporting questionnaire (SRQ-20). A-List of Threatening Experiences and the Oslo social support instruments were used to identify the factors. We used bivariate and multivariable binary logistic regressions to identify factors associated with common mental disorders. Statistical significance was declared at P-value < 0.05.

**Results:**

The prevalence of common mental disorders over the last four weeks was found to be 29.7% with 95% of confidence interval (CI) (26.4–33.1). After adjusting possible confounders, female sex, [AOR = 2.47, 95% CI (1.68, 3.62)], poor social support [AOR = 2.34, 95% CI (1.50, 3.64)], family history of mental illness [AOR = 2.15, (1.32–3.51)], rural resident [AOR = 2.01, 95% CI (1.35, 3.01)], current use of khat [AOR = 1.69, 95% CI (1 0.07, 2.64)] current use of tobacco (AOR = 1.71, 95% CI (1.04–2.84) and unemployment [AOR = 1.762, 95% CI; 1.193, 2.602)] were significantly associated with common mental disorders.

**Conclusion:**

The prevalence of common mental disorders was high, especially in Female sex, current substance use (khat chewing (leaves) and tobacco smoking), unemployment, rural residence, family history mental illness, and poor social support are the main determinants of common mental disorders. Early detection and appropriate intervention for common mental disorders in the community level should be promoted. Governmental strategies should be focused on implementing substance rehabilitation centers to treat Khat and tobacco might be helpful to minimize the burden of CMD in Ethiopia.

## Background

Mental disorder is a global public health concern. According to the recent global data, one in five individuals were suffered with common mental disorders in the last year and 29.2% of individuals had episodes of common mental disorders (CMDs) in their lifetime [1]. The other WHO report showed, 14% of the total burden of disease was accounted for CMD. It is estimated to be the first leading cause of global burden of disease by the year 2030 [2]. Mental disorders were the leading cause of years lived with disability (YLDs) and disability-adjusted life years (DALYs) worldwide, it contributed to 32.4% of YLDs and 13.0% of DALYs [3]. Mental disorders accounted for 11.1% of the total burden of diseases in low and middle-income countries due to low mental health attention, poverty, lower education, HIV/AIDS, conflict, disaster, and gender disadvantage in the region [4].

In sub-Saharan Africa, the total burden of mental and substance use problems is estimated to rise by 130%, and approximately 20 million YLDs to 45 million YLDs could be experienced in the year 2050. This estimation significantly affected health and productivity in the region [5].

Different studies showed that the prevalence of common mental disorders varied across the world. In Great Britain (England, Wales, and Scotland), 24.6% of study participants were suffered from CMD [6]. A study conducted in Brazil reported that the magnitude of CMD was 29.3% [7]. Another data in China showed that approximately 34.4% of study participants had CMD [8].

In Africa, the estimated prevalence of CMD in Tanzania ranged from 24–28.8% [9, 10]. And the higher prevalence of CMD was reported from 10.3% in Kenya to 27% in South Africa [11, 12]. In Ethiopia, different community-based research was carried out and the magnitude of CMD ranged from 14.9 to 33.6% [13–15]. Low monthly income, poor social support, presence of chronic diseases, female sex, current cigarette smoking, lower education, unemployment, the experience of stressful life events, widowed and separated were all identified as a risk factors of common mental disorders in several studies [13–17]. Where the burden of common mental disorder is higher in low- and middle-income countries, but the health care service for individuals who are suffering from mental health problems at the primary health care setting is extremely limited. Most people in Ethiopia use traditional methods for treating mental illness and seek help from their families (local communities) for their problems. Therefore, assessing the prevalence of common mental disorders and associated factors at the community level is important for early intervention and the reduction of the burden of CMDs and to improve the victims’ quality of life.

## Methods

### Study design and period

A community-based cross-sectional study was used to determine the prevalence of common mental disorders and their associated factors among residents of south the Gondar zone from May 3 to June 3, 2018.

### Study area

Debre Tabor town is the capital city of the south Gondar zone which is 666 km far from Addis Ababa (the capital city of Ethiopia) and 99 km far from Bahir Dar (the capital city of Amhara region). The zone is divided into 15 districts. According to the 2007 population census report; the total population size of South Gondar is estimated at 2,051,738. From those, 1,041,061 are men and 1,010,677 women. From the total population, 9.53% of the population is urban residents. This zone had seven primary and one referral hospital providing health services during the data collection period.

### Source population

All adults whose age is 18 years and above in south Gondar zone, Northwest, Ethiopia.

### Study population

Adults who were living in south Gondar zone selected districts/woredas during the study period.

### Sampling procedure and sample size determination

**Fig.1:**
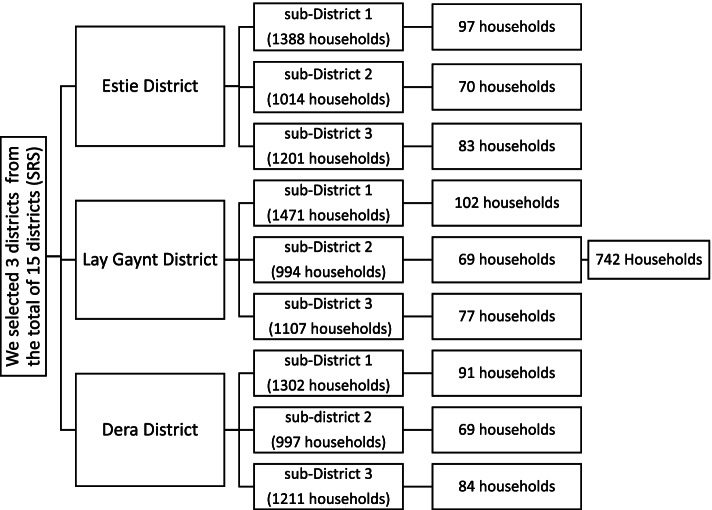
Schematic presentation of sampling procedure in adult residents of south Gondar zone, northwest Ethiopia,2018. *n*=742

We used a multistage sampling technique to select 731 participants. From 15 districts/woredas we randomly select 3 districts by simple random sampling. After selecting the districts, we selected three sub-districts/kebeles in each of the selected districts. To reach households of each sub-districts, simple random sampling was employed. In each of the areas, household lists were obtained from sub-district offices and health extension workers. We proportionally allocated the sample size to each district and further to the sub-district. The selected household members were further sorted for interviews. In the case of more than one adult study participant in a household, we selected one of them by lottery method (Fig. 1).

We determined the sample size by using the single population proportion formula by taking the prevalence of common mental disorders 32.4% [15] with a 5% margin of error, and 95% confidence interval. We added a 10% nonresponse rate and considering the design effect of 2, the final sample size was 742.

### Inclusion and exclusion criteria

All individuals whose age was 18 years and above were included in the study during the data collection period. Individuals who were seriously ill and unable to communicate were excluded from participating in the study.

### Study variables

#### Dependent variable

**Common mental disorders** (Yes = 1, No = 0).

#### Independent variables

**Socio-demographic characteristics**: age, educational status, sex, ethnicity, marital status, employment status, and residence.

**Clinical factors**: family history of mental illness, and comorbid medical/surgical illness.

**Psychosocial factors**: social support and stressful life events.

**Behavioral factors**: ever and current use of substances (khat, alcohol, and cigarette).

### Operational definitions

**Common mental disorders:** assessed using self-reporting questionnaire (SRQ-20), a score of 6 or more considered having CMD [18].

**Social support:** measured using Oslo social support scale (OSS-3), a score of 3–8, 9–11, and 12–14 categorized as poor, moderate, and strong social support respectively [19].

**Individual stress levels:** were measured using a 12 item List of Threatening Experiences (LTE), if a study participant experienced one or more stressful life events for the last six months [20].

**Current and ever use of substance:** assessed using adopted alcohol, smoking, and substance involvement screening test (ASSIST). If a study subject using at least one of the specified substances in the last 3 months and lifetime considered as current and ever of use substance respectively [21].

**Comorbid physical illness**: to assess comorbid physical illness, study subjects were asked “Did you have any comorbid physical or surgical illness?” with a response of “Yes” considered having comorbid medical/surgical illness.

**Family history of mental illness:** was measured by asking “Did you have a family history of mental illness?” with a response of “Yes” considered having a family history of mental illness.

### Data collection procedures and instruments

Data were collected by face-to-face interviews using a semi-structured questionnaire that contained socio-demographic, social support, clinical factors, and substance-related factors.

Common mental disorders were assessed using a self-reporting questionnaire (SRQ-20). A 20-item mental disorder screening instrument was developed by WHO to screen CMD. The tool measures depression, anxiety, and psychosomatic symptoms, known as CMD. Each item of SRQ has rated on a two-point scale (yes/no), “0” indicates the absence of the symptom and “1” indicates the presence of the symptom. A score of 6 or more in the self-reporting questionnaire was considered as having common mental disorders in the last one month. The tool was validated in low-and-middle-income countries with a sensitivity 78.6% and a specificity 81.5% [18, 22].

Social support was measured by using three items of the Oslo social support scale (OSS-3). The sum score of Oslo social support ranges from 3 to 14 with higher score indicating strong support and lower score indicating lower support. The instrument categorized as scoring of 12–14 considered “strong support”, the score of 9–11 “moderate social support” and score of 3–8 “poor social support” [19].

Individual stress levels were measured by using a 12 item List of Threatening Experiences (LTE). The tool measures the individual level of stress for the last six months. Each item of LTE has rated on a two-point scale (yes/no), “1” indicates the presence and “0” indicates absence of stressful life events over the last six months [20]. To assess the current and ever use of substance-using adopted alcohol, smoking, and substance involvement screening test (ASSIST). If a study participant using at least one of the specified substances in the last 3 months and lifetime considered as current and ever of use substance respectively [21].

### Data quality control issues

We recruited degree holder psychiatry professionals for data collection and supervised by Master holder psychiatry professionals. The training was given on the data collection instrument and sampling procedure. Additionally, the questionnaire was designed in English and translated to Amharic language (local language). The supervision was held regularly during the data collection period.

### Data processing and analysis

Data were entered into Epi-data 3.1 after checking completeness and consistency and then exported to SPSS—version 20 for analysis. Factors associated with CMDs were selected during bivariate analysis with a value of p ≤ 0.2. In multivariable regression analysis variables with P-value, less than 0.05 at 95% confidence interval with its adjusted odds ratio were considered as statistically significant.

### Ethical clearance

Ethical approval was obtained from the ethical review committee of Debre Tabor University. Ethical clearance was also obtained from the ethical review committees of the University (Ref. No. DTU/RE/1/P5/2017). Permission was obtained from the respective district administration.

## Results

A total of 731 respondents were interviewed with a response rate of 98.5%. The majority of the respondents, 386 (52.8%) were male; two hundred forty-two (40.2%) were in the age range of 26–40 years; four hundred (54.7%) were married; two hundred sixty-eight (36.7%) were grade 9–12; almost half (50.6%) of respondents were residing in urban areas; 693 (94.8%) were Orthodox Christian, and seven hundred eighteen (98.2%) Amhara by ethnicity. Regarding occupation, more than half (73.6%) were employed (Table 1).

**Table 1:** Socio-demographic characteristics of respondents in south Gondar zone, northwest Ethiopia, 2018

Variable	Category	Frequency	Percentage
Age	18–25	203	27.8%
	26–40	294	40.2%
	> = 41	234	32.0%
Sex	Male	386	52.8%
	Female	345	47.2%
Ethnicity	Amhara	719	98.4%
	other	12	1.6%
Educational status	Unable to read and write	98	13.4%
	1–8 grade	203	27.8%
	9–12 grade	268	36.7%
	Diploma & above	162	22.1%
Religion	Orthodox	693	94.8%
	Muslim	38	5.2%
Marital status	Single	262	35.8%
	Married &living together	400	54.7%
	Separated	20	2.7%
	Divorced	30	4.1%
	Widowed	19	2.6%
Residence	Rural	361	49.4%
	Urban	538	50.6%
Employment status	Non-employed	193	26.4%
	Employed	342	73.6%

### Distribution of clinical and psychosocial factors

Of the respondents, 100 (13.7%) had a family history of mental illness, sixty-five (8.9%) had a comorbid physical illness. Regarding social support, nearly one-third (31.9%) had poor social support and 311(42.5%) had strong social support (Table 2).

**Table 2:** Distribution clinical and psychosocial factors of respondents in south Gondar zone, northwest Ethiopia, 2018

Variable	Category	Frequency	Percentage
Social support	poor	233	31.9
	moderate	187	25.6
	strong	311	42.5
Family history of mental illness	yes	100	13.7
	no	631	86.3
Co-morbid medical/surgical illness	yes	65	8.9
	no	666	91.1

### Substance use characteristics

**Fig. 2:**
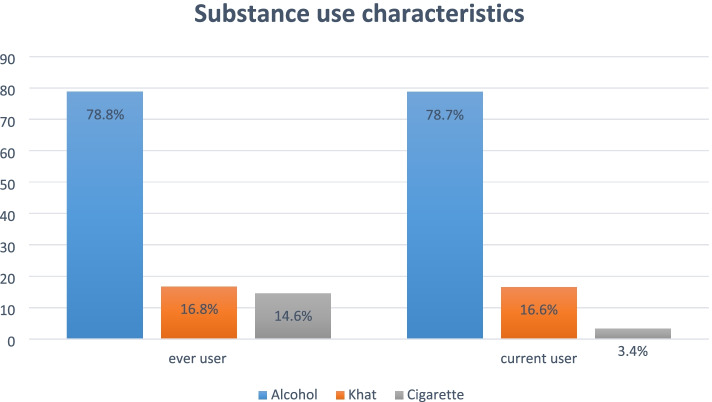
Substance use characteristics among residents of South Gondar Zone, Northwest Ethiopia, 2018. (*n*=731)

Regarding substance-related factors, more than three-fourths of the participants (78.8%) were consumed alcohol, and 576(78.7%) drinking alcohol at the moment; 121(16.6%) were using khat (leaves), and 107(3.4%) were smoked currently (Fig. 2).

### Prevalence of common mental disorders

The prevalence of the common mental disorders among participants was 29.7% (95% CI 26.4, 33.1).

### Factors associated with common mental disorders

To determine the association of independent variables with CMDs, bivariate, and multivariate binary logistic regression analyses was carried out. In the bivariate analysis factors associated with CMDs at a *P*-value, less than 0.2 were entered into the multivariable logistic regression model to control confounding effects.

The result of the multivariate analysis showed that female sex, current use of khat (chewing leaves), rural residence, social support, and current use of smoking, unemployment, and family history of mental illness was significantly associated with common mental disorders at a *p*-value less than 0.05.

Individuals who use khat currently were 1.7 times more likely to have common mental disorders than individuals who didn’t use khat currently (AOR = 1.69, 95% CI: 1 0.07, 2.64). Respondents who had poor social support were 2.3 times more likely to develop CMDs compared with those who had strong social support (AOR = 2.34, 95% CI:1.50, 3.64). Similarly, unemployment had 1.7 times more likely to have common mental disorders when compared to employed individuals (AOR = 1.762, 95% CI; 1.193, 2.602). The likelihood of developing CMDs was 2.1 times higher among respondents who had a family history of mental illness compared with those who had no family history of mental illness (AOR = 2.15, 95% CI:1.32–3.51). The female sex was 2.4 times more likely to develop CMDs compared with the male sex (AOR = 2.47, 95% CI (1.68, 3.62). Participants residing in rural were 2.0 times high likely to have CMDs than participants who live in urban (AOR = 2.01, 95% CI: 1.35, 3.01). Participants smoking at the moment were 1.7 times high likely for CMDs compared to ever use of tobacco (smoking) (AOR = 1.71, 95% CI: 1.04–2.84) (Table 3).

**Table 3:** Bivariate and Multivariable analysis of common mental disorders among respondents in south Gondar zone, northwest Ethiopia, 2018

Variables	Category	Common mental disorders		COR (95% CI)	AOR (95% CI)
		**No**	**Yes**		
Sex	Male	295	91	1	1
	Female	219	126	1.86(1.353–2.572)	2.46(1.683–3.620)*
Job status	Employed	396	142	1	1
	Non-employed	118	75	1.77(1.253–2.508)	1.76(1.193–2.602)*
Social support	Strong	244	67	1	1
	Moderate	140	47	1.22(0.798–1.874)	1.32(0.832–2.095)
	Poor	130	103	2.88(1.985–4.194)	2.33(1.499–3.638)*
Current use of khat	No	442	168	1	1
	Yes	72	49	1.79(1.195–2.682)	1.69(1.086–2.642)*
Current use tobacco (smoking)	No	454	170	1	1
	Yes	60	47	2.09(1.374–3.185)	1.71(1.035–2.840)*
Family history of mental illness	No	463	168	1	1
	Yes	51	49	2.64(1.723–4.070)	2.15(1.320–3.506)*
Residence	Urban	278	92	1	1
	Rural	236	125	1.60(1.162–2.205)	2.01(1.349–3.006)*
Comorbid physical illness	No	37	28	1	1
	Yes	477	189	1.91(1.137–3.209)	0.79(0.425–1.502)

^*^ *P* < 0.05, *COR* Crude Odds Ratio, *AOR* Adjusted Odds Ratio

## Discussion

Common mental illnesses are a public health concern, that have negative impact on physical health, psychosocial and economic consequences. In this study, the prevalence of common mental disorders was 29.7% (95% CI: 26.4, 33.1). Our finding was consistent with previous studies conducted in Ethiopia, 32.4% [15], in South Africa 27% [11], in Brazil 29.9% [7], in Santiago, Chile 26.7% [23], and in Great Britain 27.2% [24]. However, the finding of this study was higher than the Previous Ethiopian studies which range from 14.9% to 22.7% [13, 25, 26], in Kenya (10.3%-10.8%) [12, 16], in Tanzania 4.1% [10], in Taiwan 23.8% [27], and in southeast London 24.6% [6]. Conversely, this finding was lower than the 33.6% noted in Jimma town, Southwest Ethiopia [14], 33.9% in India [28], 52.5% in rural Bangladesh [29], and 34.4% in China [8]. The possible reason for this difference might be the use of different instruments and cut-off points to measure common mental disorders. That is, the other studies used a revised clinical interview schedule (CIS-R), Kessler 10 item questionnaire, and a general health questionnaire (GHQ-12), while we utilized SRQ-20. The other variation might be the number of participants in the study. That is, a study done in Bangladesh, 2425 study participants were included, in southeast London 1968, in southern India 327, and in China 3031 participants were included. However, this finding showed that the magnitude of common mental disorders is still increased in Ethiopia. This implies that different stakeholders at different levels should have a plan for early intervention, and treatment accessibility at the level of primary health care settings.

This study showed that the female sex had a 2.4 times higher risk of CMDs compared with the male sex. This might be due to various of factors, including hormonal differences, the effects of childbirth, psychosocial stressors (high household responsibility), as a result of physical abuse, and behavioral models of learned helplessness. In addition to this low socio-economic status of females might have predisposed them to a higher risk for common mental disorders. This finding was supported by studies carried out in India [30], Brazil [7], Chile [23], rural Bangladesh [29], South Africa [31], Kenya [12], and previous Ethiopian studies [14, 15]. There was a statically significant association between common mental disorders and current tobacco use (smoking). Smoking has its own effect on physical and psychological functioning. The effect of nicotine smoking can lead to dependency, stigma, and behavioral influence and reduce the economic status of the participant. This finding was supported by previous epidemiological studies conducted in Ethiopia [13, 14].

Regarding employment status, common mental disorders were significantly associated with unemployment. The significant association between unemployment and CMDs in the present study was similarly reported in previous community-based studies carried out in Ethiopia [26, 32], Chile [23], Taiwan [27], Tanzania [10], and South Africa [11]. The possible explanation might be unemployment is a major risk of a serious social problem that led to the loss of income, increases the risk of poverty, and affect overall health status. Although, unemployment is a predictor of change in social position, especially a change in family role, and is a serious consequence for mental illness [33–37].

In the present study, participants with poor social support were more likely to have common mental disorders when compared with participants with strong social support. This finding was consistent with a previous Ethiopian study [32]. This is might be due to poor experience in social relationships, social related and psychological support from the community, neighborhood people, and relatives can lead to common mental disorders. Participants who live in rural areas were 2 times more likely to have common mental disorders compared with individuals who live in urban areas. The finding is in line with other studies in China [8], and Nigeria [38]. Common mental disorders were significantly higher among those who reported currently chewing khat as compared to those who didn’t chew khat currently. This might be due to the impact of khat on physical and psychological function. The psychosocial effect of khat chewing depends on its capacity to led to dependency or addiction and to the specific physical and behavioral effects including socio-economic effects for individuals might be led to common mental disorders. This finding is supported by previous studies [13–15]. Family history of mental illness was significantly associated with common mental disorders. Parental history of mental illness might increase the risk of CMDs in the offspring through different reasons like transmission of genetic factors. This finding is similar to studies carried out in Ethiopia [25, 26].

### Limitation of the study

Our study design prevented us from concluding the cause-and-effect relationships of the associations. recall bias might be also one of the other limitations. Since the data collection method was a face-to-face interview which might lead individuals to respond in socially acceptable ways during the process, especially in cases of substance-related questions.

## Conclusion

The magnitude of common mental disorders was found to be high. Female sex, current substance use (khat chewing (leaves) and tobacco smoking), unemployment, rural residence, family history mental illness, and poor social support were significantly associated with common mental disorders. Therefore; we recommend common mental disorders (depression, anxiety, and somatic symptom) focused on early regular screening by trained health professionals and linkage with mental health service providers. It is necessary to give emphasis to individuals with a family history of mental illness, women, and a history of mental illness. Governmental strategies should be focused on implementing substance rehabilitation centers to treat Khat and tobacco might be helpful to minimize the burden of CMD in Ethiopia.

## Data Availability

All data are available in the manuscript.

## Abbreviations

CMD: Common mental disorders
SRQ: Self-reporting questionnaire
DALYs: Disability Adjusted Life Years
YLD: Years lived with disability
OSS-3: Social Support Scale
ASSIST: Alcohol, smoking, and substance involvement screening test
LTE: List of Threatening Experiences

## References

[1] Steel Z, Marnane C, Iranpour C, Chey T, Jackson JW, Patel V (2014). The global prevalence of common mental disorders: a systematic review and meta-analysis 1980–2013. Int J Epidemiol.

[2] Organization WH. The world health report 2003: shaping the future: World Health Organization; 2003.

[3] Vigo D, Thornicroft G, Atun R (2016). Estimating the true global burden of mental illness. The Lancet Psychiatry.

[4] Patel V (2007). Mental health in low-and middle-income countries. Br Med Bull.

[5] Charlson FJ, Diminic S, Lund C, Degenhardt L, Whiteford HA (2014). Mental and substance use disorders in sub-Saharan Africa: predictions of epidemiological changes and mental health workforce requirements for the next 40 years. PLoS ONE.

[6] Weich S, Lewis G (1998). Poverty, unemployment, and common mental disorders: population based cohort study. BMJ.

[7] Rocha SV, Almeida MMGD, Araújo TMD, Virtuoso Júnior JS (2010). Prevalence of common mental disorders among the residents of urban areas in Feira de Santana Bahia. Revista Brasileira de Epidemiologia.

[8] Zhong B-L, Liu T-B, Chan S, Jin D, Hu C-Y, Dai J (2018). Common mental health problems in rural-to-urban migrant workers in Shenzhen, China: prevalence and risk factors. Epidemiol Psychiatr Sci.

[9] Uriyo JG, Abubakar A, Swai M, Msuya SE, Stray-Pedersen B (2013). Prevalence and correlates of common mental disorders among mothers of young children in Kilimanjaro region of Tanzania. PLoS One.

[10] Jenkins R, Mbatia J, Singleton N, White B. Common mental disorders and risk factors in urban Tanzania. Int J Environ Res Public health. 2010;7(6):2543-58. 10.3390/ijerph7062543. 10.3390/ijerph7062543 PMC2905566 20644689

[11] Havenaar JM, Geerlings MI, Vivian L, Collinson M, Robertson B (2008). Common mental health problems in historically disadvantaged urban and rural communities in South Africa: prevalence and risk factors. Soc Psychiatry Psychiatr Epidemiol.

[12] Jenkins R, Othieno C, Ongeri L, Sifuna P, Ongecha M, Kingora J (2015). Common mental disorder in Nyanza province, Kenya in 2013 and its associated risk factors–an assessment of change since 2004, using a repeat household survey in a demographic surveillance site. BMC Psychiatry.

[13] Hunduma G, Girma M, Digaffe T, Weldegebreal F, Tola A (2017). Prevalence and determinants of common mental illness among adult residents of Harari Regional State, Eastern Ethiopia. Pan Afr Med J.

[14] Kerebih H, Soboka M (2016). Prevalence of common mental disorders and associated factors among residents of Jimma town. South West Ethiopia Population.

[15] Yimam K, Kebede Y, Azale T (2014). Prevalence of common mental disorders and associated factors among adults in Kombolcha Town. Northeast Ethiopia J Depress Anxiety S.

[16] Jenkins R, Njenga F, Okonji M, Kigamwa P, Baraza M, Ayuyo J (2012). Prevalence of common mental disorders in a rural district of Kenya, and socio-demographic risk factors. Int J Environ Res Public Health.

[17] Tareke M, Birehanu M, Amare D, Abate A (2020). Common mental illness among epilepsy patients in Bahir Dar city, Ethiopia: a cross-sectional study. PLoS One.

[18] Youngmann R, Zilber N, Workneh F, Giel R (2008). Adapting the SRQ for Ethiopian populations: a culturally-sensitive psychiatric screening instrument. Transcult Psychiatry.

[19] Dalgard OS, Dowrick C, Lehtinen V, Vazquez-Barquero JL, Casey P, Wilkinson G (2006). Negative life events, social support and gender difference in depression. Soc Psychiatry Psychiatr Epidemiol.

[20] Brugha T, Bebbington P, Tennant C, Hurry J (1985). The List of Threatening Experiences: a subset of 12 life event categories with considerable long-term contextual threat. Psychol Med.

[21] Henrique IFS, De Micheli D, Lacerda RBD, Lacerda LAD, Formigoni MLODS (2004). Validation of the Brazilian version of alcohol, smoking and substance involvement screening test (ASSIST). Rev Assoc Med Bras.

[22] Netsereab TB, Kifle MM, Tesfagiorgis RB, Habteab SG, Weldeabzgi YK, Tesfamariam OZ (2018). Validation of the WHO self-reporting questionnaire-20 (SRQ-20) item in primary health care settings in Eritrea. Int J Ment Heal Syst.

[23] Araya R, Rojas G, Fritsch R, Acuña J, Lewis G (2001). Common mental disorders in Santiago, Chile: prevalence and socio-demographic correlates. Br J Psychiatry.

[24] Weich S, Sloggett A, Lewis G (1998). Social roles and gender difference in the prevalence of common mental disorders. Br J Psychiatry.

[25] Alem A, Kebede D, Woldesemiat G, Jacobsson L, Kullgren G (1999). The prevalence and socio-demographic correlates of mental distress in Butajira. Ethiopia Acta psychiatrica scandinavica.

[26] Mekonnen E, Esayas S. Correlates of mental distress in Jimma town, Ethiopia. Ethiopian journal of health sciences. 2003;13(1).

[27] Fu TST, Lee CS, Gunnell D, Lee WC, Cheng ATA (2013). Changing trends in the prevalence of common mental disorders in Taiwan: a 20-year repeated cross-sectional survey. The Lancet.

[28] Pothen M, Kuruvilla A, Philip K, Joseph A, Jacob K (2003). Common mental disorders among primary care attenders in Vellore, South India: nature, prevalence and risk factors. Int J Soc Psychiatry.

[29] Islam FMA (2019). Psychological distress and its association with socio-demographic factors in a rural district in Bangladesh: a cross-sectional study. PLoS One.

[30] Patel V, Araya R, De Lima M, Ludermir A, Todd C (1999). Women, poverty and common mental disorders in four restructuring societies. Soc Sci Med.

[31] Herman AA, Stein DJ, Seedat S, Heeringa SG, Moomal H, Williams DR (2009). The South African Stress and Health (SASH) study: 12-month and lifetime prevalence of common mental disorders. S Afr Med J.

[32] Solomon A, Mihretie G, Tesfaw G (2019). The prevalence and correlates of common mental disorders among prisoners in Addis Ababa: an institution based cross-sectional study. BMC Res Notes.

[33] Karanikolos M, Mladovsky P, Cylus J, Thomson S, Basu S, Stuckler D (2013). Financial crisis, austerity, and health in Europe. The Lancet.

[34] Marmot MG, Bell R (2009). How will the financial crisis affect health?. Bmj.

[35] Gallagher S, Sumner RC, Muldoon OT, Creaven A-M, Hannigan A (2016). Unemployment is associated with lower cortisol awakening and blunted dehydroepiandrosterone responses. Psychoneuroendocrinology.

[36] Daly M, Delaney L (2013). The scarring effect of unemployment throughout adulthood on psychological distress at age 50: Estimates controlling for early adulthood distress and childhood psychological factors. Soc Sci Med.

[37] Booker CL, Sacker A (2012). Psychological well-being and reactions to multiple unemployment events: adaptation or sensitisation?. J Epidemiol Community Health.

[38] Amoran O, Lawoyin T, Lasebikan V (2007). Prevalence of depression among adults in Oyo State, Nigeria: A comparative study of rural and urban communities. Aust J Rural Health.

